# Cities, Country Towns, and Villages, and Proprietors of Mail and Stage Coach Establishments

**Published:** 1818

**Authors:** James Carver


### CITIES,
### COUNTRY TOWNS, AND VILLAGES,
AND
### PROPRIETORS
OF
### *Mail and Stage Coach Establishments,*

Will find it an object of great commercial, as well as domestic importance to their own interests in obtaining smiths, and inviting them to receive instruction, and to establish themselves on their different lines. In a political point of view, also this establishment may become of great importance, which must be sufficiently manifest; so fully of late, was the utility of it estimated by the government of England, Ireland, and Scotland, in their *military* and *mail* department, that an annual grant of 15,000*l.* has been voted for the support of the Veterinary College, and for the education of pupils for the improvement of the shoeing art.

Proprietors of such establishments will therefore find it their interest to consult Dr. Carver, or the following gentlemen.

Happily for the profession itself, and much more happily for the community at large, that similar advantages are beginning to make some progress by the aforesaid establishment; and it is to be hoped that the time is not far distant, when it will be further honoured by a legislative contribution, under which predictive ray of reformation, part of the present generation may probably not only derive future advantage, but live to see the former system rescued from the ignorance and barbarity, by which it has long been disgraced in this part of the world. The following gentlemen, therefore, seeing the ignorance and incompetency of farriers, and others, who have hitherto practised on the diseases of horses in this city, (and to remedy this, and meet the *evil*, in the most effectual manner), have cheerfully stepped forward to sanction and support it.

*The medical patrons are,*

Dr. Chapman, Professor of Anatomy, M. M.

Dr. Hewson, Professor of Comparative Anatomy.

*The patrons friendly to Dr. Carver's New Establishment are,*

John Tomlinson,

Mr. Morrison,

Charles Watson,

Mr. Schlatter,

Condy Raguet,

John Vaughan,

W. Wurts,

M. Wurts,

Roberts Vaux;

Reuben Haines,

Jeremiah Warder,

William Sansom,

Dr. Mease,

Alexander Henry,

Wilson Hunt.

To whom proprietors of the above-mentioned establishments, city or country smiths may apply for an introduction to Dr. Carver, and who, on receiving the same, shall be supplied with models of all the patent college Shoes, Hammers, Countersink Nails, Punches, Fullers, Instruments, and Drawing Knives, &c. The Butteris must be abolished for the use of the Drawing Knife, and smiths being instructed in the use of that instrument, by Dr. Carver, will be entitled to shoe, on the above principle, for no less than two dollars a set. Every gentleman travelling to different parts of the United States, and desirous of giving encouragement to the establishment for disseminating this useful and necessary branch of domestic science, will be entitled to a set of the Model Patent Shoes; and on bringing his horse or horses, to the forge for examination by Dr. Carver, he will be instructed in their use and application.

Country smiths, respectable young men desirous of emulating in this branch of the Veterinary Art, as well as to learn the surgical, operative parts of the profession, will do well to apply before the ensuing winter. And every person being so instructed, and found qualified, will receive a proper certificate, signed by the medical patrons, gentlemen and professor of the aforesaid establishment, as being better qualified to practise with advantage to themselves and to the public.

*Note.*—Dr. Carver being a member of the London Veterinary Medical Society, and having been instructed in the art of compounding Veterinary Medicine at the College *Pharmacopœia*, has also established a small Laboratory at his own house, where gentlemen, travelling to the different parts of the United States, may be supplied with what Medicines they may want. City and country druggists, desirous of retailing, an arrangement of the above ready prepared Medicines, may be supplied also. Each article containing a regular practical treatise on the complaint intended to be removed.

